# A Case Report of a Giant Placental Chorioangioma: Prenatal Diagnosis and Perinatal Outcome

**DOI:** 10.7759/cureus.91642

**Published:** 2025-09-05

**Authors:** Elif Empliouk, Apostolos Sidiropoulos, Christina Yfanti, Evaggelia Tsakmaki, Liountmila Romanidou, Konstantina Tsitsila, Nikoleta-Dimitra Savvidou, Effrosyni Styliara, Alexandros Traianos, Stefanos Flindris, Alexandros Sotiriadis

**Affiliations:** 1 2nd Department of Obstetrics and Gynecology, Aristotle University of Thessaloniki, School of Medicine, Thessaloniki, GRC; 2 Department of Pathology, General Hospital of Thessaloniki Ippokrateio, Thessaloniki, GRC; 3 Department of Radiology, University General Hospital of Ioannina, Ioannina, GRC

**Keywords:** amnioreduction, chorioangioma, doppler ultrasound, fetal anemia, polyhydramnios

## Abstract

Chorioangiomas are benign placental vascular tumors, most commonly detected during antenatal ultrasound screening. While small lesions are often clinically insignificant, giant chorioangiomas defined as those exceeding 4 cm in diameter can be associated with serious fetal and maternal complications, including fetal anemia, thrombocytopenia, fetal heart failure, fetal hydrops, placentomegaly, polyhydramnios, maternal mirror syndrome, fetal demise, and neonatal death. Doppler ultrasound and magnetic resonance imaging aid in differentiating these tumors from other placental lesions. Treatment is tailored according to gestational age, tumor size, and associated complications. Small asymptomatic lesions may require only routine surveillance, whereas large or symptomatic chorioangiomas can necessitate interventions such as intrauterine transfusion, amnioreduction, or more advanced procedures like fetoscopic-assisted ablation or embolization. We present the case of a 30-year-old primigravida (G1P0) diagnosed with a large placental chorioangioma and polyhydramnios at 28 weeks of gestation during a routine growth ultrasound, who was referred to our tertiary care center for further management. The patient underwent two ultrasound-guided amnioreduction procedures for symptomatic polyhydramnios. At 31 weeks of gestation, she experienced preterm premature rupture of membranes (PPROM), followed by spontaneous labor, and subsequently delivered via cesarean section. A preterm neonate was born with favorable immediate postnatal outcomes. This case underscores the importance of early diagnosis and individualized, timely management in reducing fetal and maternal morbidity and mortality associated with large placental chorioangiomas. By detailing the diagnostic challenges and therapeutic decisions in a case complicated by polyhydramnios, this report aims to contribute meaningful insight to the existing literature.

## Introduction

Chorioangiomas are well-circumscribed placental vascular tumors that protrude into the amniotic cavity [1]. The majority are small (less than 4 cm), remain undiagnosed, and are asymptomatic. In contrast, large chorioangiomas, typically those exceeding 4 cm, are rare, with an estimated prevalence ranging from one in 9,000 to one in 50,000 pregnancies [2]. Complications are primarily associated with large chorioangiomas, with increased risk for fetal morbidity such as fetal anemia and thrombocytopenia due to sequestration of red blood cells and platelets by the tumor [1,2]. Additionally, large chorioangiomas can lead to fetal heart failure, fetal hydrops, fetal distress, placentomegaly, polyhydramnios, and maternal mirror syndrome (generalized fluid overload and preeclampsia) [3-5]. Severe polyhydramnios may cause maternal discomfort, uterine overdistention, and an increased risk of preterm labor and preterm premature rupture of membranes (PPROM) [6].

Although the precise etiology of a chorioangioma is not well understood, it is thought to arise from abnormal proliferation of blood vessels at various stages of development within a fibrous stroma [7]. Reported perinatal mortality for symptomatic cases is between 30% and 40% [8]. Prenatal diagnosis of placental chorioangioma relies on gray-scale sonography, color Doppler ultrasonography, and magnetic resonance imaging (MRI) [5]. On ultrasound, chorioangiomas appear as hypoechoic, rounded, well-circumscribed placental masses with homogeneous or heterogeneous internal architecture located on the fetal surface of the placenta [9-11]. Doppler imaging is valuable for assessing vascular characteristics and distinguishing chorioangiomas from other lesions such as hematomas, partial hydatidiform moles, teratomas, metastatic tumors, and leiomyomas; increased blood flow on color Doppler is a hallmark feature [5].

Doppler evaluation of the fetal middle cerebral artery peak systolic velocity (MCA PSV) is used to detect fetal anemia; a PSV > +1.5 multiple of the median (MoM) is indicative of moderate-to-severe anemia [12,13]. MRI serves as a complementary tool when ultrasound findings are inconclusive or when the lesion is large. Given the potential development of anemia, monitoring is recommended every two to three weeks, and weekly after 32 weeks of gestation, to assess tumor growth, cardiac function, MCA PSV, and amniotic fluid volume [10-12]. It is estimated that approximately 50% of large chorioangiomas result in complications that may necessitate therapeutic intervention or preterm delivery [9,11].

Management of giant chorioangiomas should be individualized based on the presence and severity of associated complications, although many cases can be managed conservatively with close surveillance. Reported treatment modalities include fetoscopic or interstitial laser ablation of feeding vessels, embolization, alcohol-induced chemosclerosis, medical therapy with propranolol, and surgical ligation of affected vessels [4,10]. Amnioreduction is a recognized therapy for severe symptomatic polyhydramnios. In cases with hyperdynamic fetal circulation leading to significant anemia, intrauterine transfusion may be indicated to correct hematologic imbalance [14,15].

We report a case of a large chorioangioma located near the placental cord insertion site, complicated by polyhydramnios that required two amnioreduction procedures. The patient was admitted for comprehensive assessment and continuous observation. Delivery occurred at 31 weeks of gestation due to PPROM with spontaneous onset of labor and subclinical chorioamnionitis, resulting in the birth of a preterm neonate.

## Case presentation

**Figure 1:**
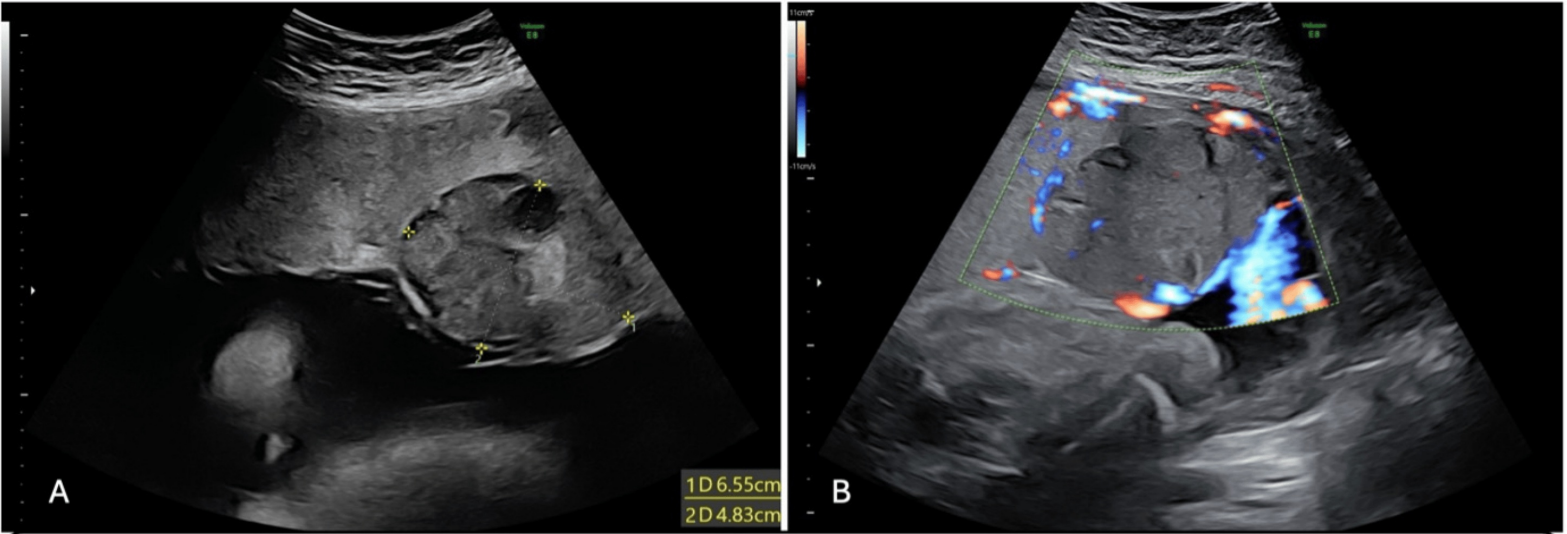
Ultrasound imaging A) Transabdominal obstetric ultrasound depicting gray-scale image of a chorioangioma. A well-defined mass measuring 65x48 mm with heterogenous echotexture observed in the placenta; B) Color doppler image of the chorioangioma revealed a highly vascularized placental tumor protruding into the amniotic cavity.

A 30-year-old G1P0 woman was referred to our fetal-maternal medicine unit from her private obstetrician-gynecologist after a routine growth ultrasound at 28 weeks of gestation revealed placental chorioangioma and polyhydramnios. She was admitted for further evaluation and management. Her body mass index was 32.8. A prior oral glucose tolerance test had excluded gestational diabetes. Initial ultrasound showed a single live fetus with an estimated fetal weight of 1482 grams, corresponding to the 93.7th percentile for gestational age, and an increased amniotic fluid index (AFI) of 30.0 cm. A well-circumscribed mass measuring 65 × 48 mm, suggestive of a large placental chorioangioma, was identified on targeted ultrasound performed by a maternal-fetal medicine specialist (Figures 1A-1B).

There were no sonographic signs of hydrops fetalis. Doppler assessment revealed a MCA-PSV of 52.3 cm/s (1.388 MoM for gestational age, <1.5 MoM), not meeting the threshold for suspected fetal anemia. Umbilical artery Doppler demonstrated normal end-diastolic flow.

A multidisciplinary consultation with the family was undertaken to explain the pathophysiology of chorioangioma, the implications of polyhydramnios, and the risks of maternal and fetal complications, including those related to prematurity and potential need for urgent preterm delivery as well as the proposed surveillance strategy, prognosis, and management options, ranging from conservative observation to intervention. After the initial assessment, prophylactic corticosteroids were administered in the form of dexamethasone phospshate (4 mg intramuscularly every six hours for eight doses) to accelerate fetal lung maturity. To prevent the symptomatic recurrence of polyhydramnios and to relieve maternal discomfort (and partially to reduce uterine overdistension-related preterm labor risk), ultrasound-guided amnioreduction was performed under aseptic technique. At 28+6 weeks of gestation, 1700 mL of amniotic fluid was removed. A second amnioreduction was required at 30+1 weeks due to recurrence, with an additional 1700 mL drained. Prophylactic intravenous antibiotics were administered (ceforoxime 1.5 gm thrice daily for five days) following each procedure to mitigate infectious risk.

During hospitalization, the fetus was closely monitored. Weekly Doppler assessments of MCA-PSV remained consistently below 1.5 MoM, effectively excluding clinically significant fetal anemia. Daily computerized cardiotocography (cCTG) demonstrated reassuring fetal heart rate patterns with minimal uterine activity. Atosiban (Tractocile) was chosen as the primary tocolytic agent because of its favorable maternal and fetal safety profile in the context of a highly vascular placental lesion. Additionally, a short 48-hour course of indomethacin was administered to medically decrease amniotic fluid production, with careful monitoring given the known potential effects on the fetal ductus arteriosus. Despite these interventions, there was progressive increase in both the intensity and frequency of premature uterine contractions.

At 31+3 weeks of gestation, the patient experienced PPROM, followed shortly by spontaneous onset of labor. Throughout this period, there were no clinical signs (e.g., fever, uterine tenderness) or laboratory abnormalities to raise suspicion for overt chorioamnionitis. Laboratory parameters prior to delivery are summarized in Table 1.

**Table 1:** Laboratory values with reference ranges WBC: White blood cells; CRP: C- reactive protein.

Parameter	Reference range	Values before the cesarean section
WBC, 10^3^/μL	4.5-10.5	10.4
Neutrophils, %	40-75	68
CRP, mg/L	<6	9.6

**Figure 2:**
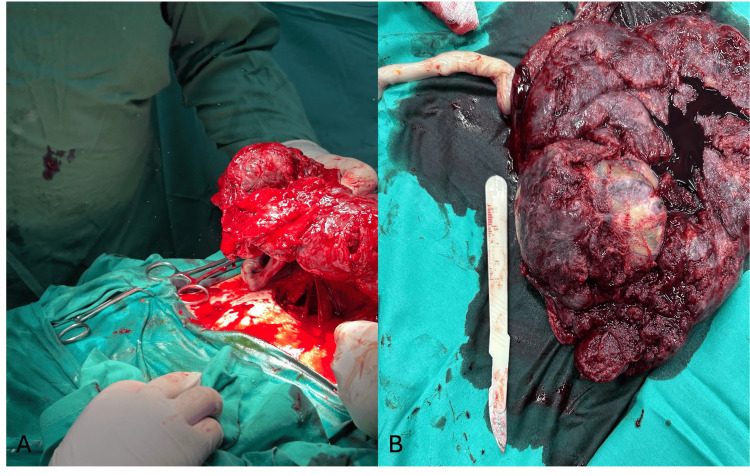
Intraoperative images A) The intraoperative image of an en bloc removal of the placenta and the chorioangioma; B) Macroscopic appearance of the placenta after the cesarean section. A 8 cm diameter mass on the fetal surface of the placenta near its edge is shown.

After further multidisciplinary discussion and given the evolving clinical picture with imminent delivery in the setting of high-risk placental pathology, a category III emergency cesarean section was performed. The intraoperative images are provided in Figures 2A-2B.

The couple had received detailed counseling regarding anticipated neonatal intensive care unit (NICU) admission, risks of postpartum hemorrhage, and potential interventions, including the possibility of peripartum hysterectomy if bleeding became uncontrollable, and informed consent was obtained.

**Figure 3:**
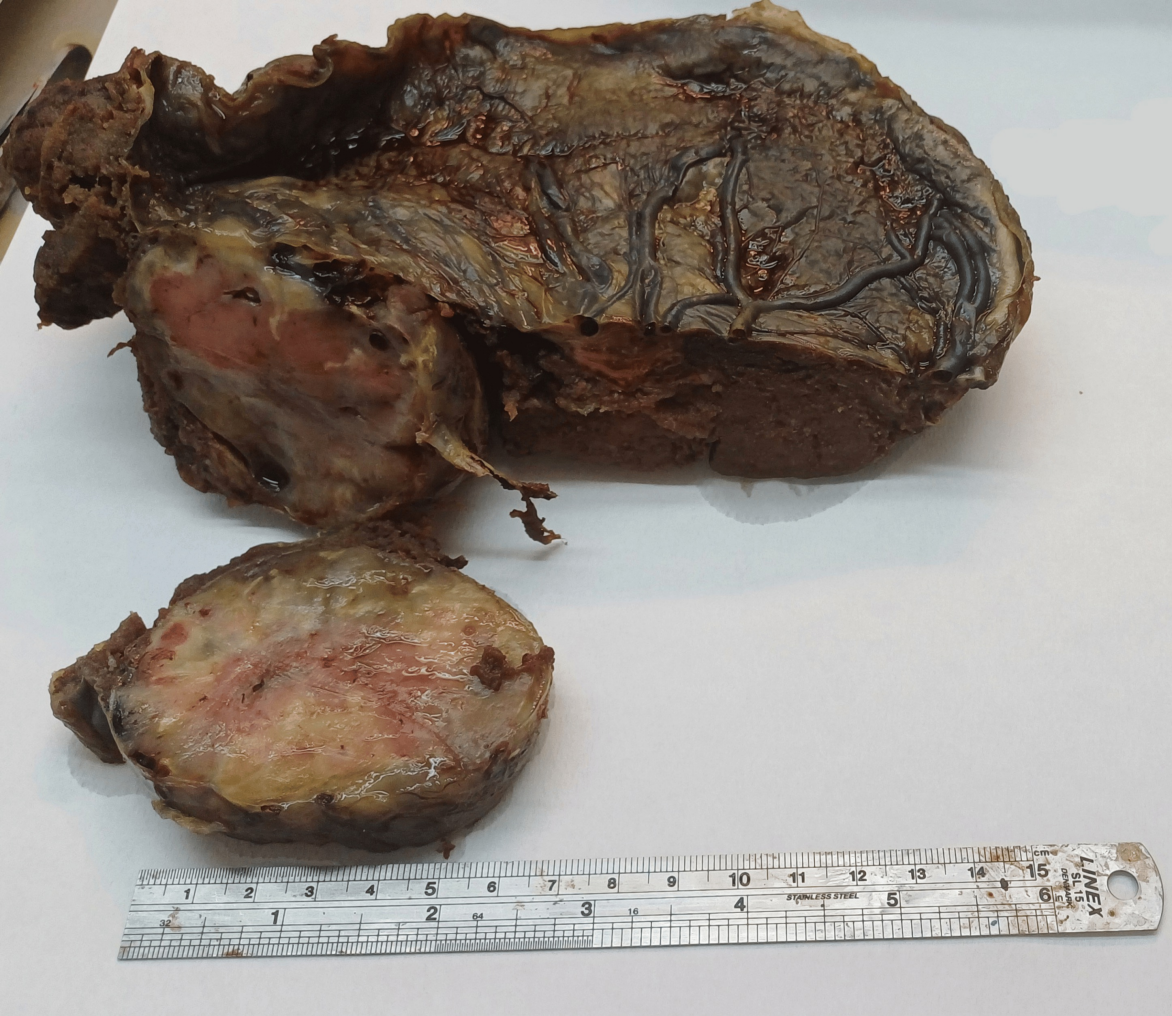
Gross specimen of the placenta with the chorioangioma

A female neonate weighing 2280 g was delivered with Appearance, Pulse, Grimace, Activity, Respiration (APGAR) scores of eight and nine at one and five minutes, respectively. The placenta with the attached chorioangioma was delivered spontaneously; total placental weight was 770 g, and the lesion measured 8x6x5 cm on gross inspection (Figure 3).

The neonate avoided intubation and required initial non-invasive respiratory support with continuous positive airway pressure (CPAP), later weaned to high-flow nasal cannula due to transient tachypnea of the newborn (TTN). Empiric intravenous antibiotic therapy with ampicillin and an aminoglycoside was initiated upon admission to the neonatal unit. The clinical course was otherwise uneventful, and the infant was discharged from the NICU on day 18 of life in good condition, without clinical or laboratory evidence of anemia or thrombocytopenia. The mother’s postoperative recovery was uncomplicated; she was discharged home on postoperative day four with cefuroxime 1.5 gm thrice daily for five days. Histopathological examination confirmed the diagnosis of giant chorioangioma and unexpectedly demonstrated histologic evidence of chorioamnionitis, suggesting a subclinical (clinically silent) intrauterine infection.

Histopathology report

Gross Findings

The disc-shaped placenta was fixed in formalin weighing 708 g (after removal of cord and membranes) with eccentric attachment of an umbilical cord of 21.5 cm in length and 1 cm in diameter containing three vessels. Beneath the cord insertion, in the subchorionic region, there was a somewhat rounded mass of reddish-brown color with a homogeneous elastic solid consistency, in places gelatinous, with yellowish areas of maximum diameter (unclear/illegible). Adjacent to this, there was a subchorionic hematoma with a maximum diameter of 3 cm. The parenchyma was hypercellular. Membranes were slightly greenish. The paraffin block included sections from rolls of membranes and cord, the mass, and full-thickness sections from the parenchyma (Figure 3).

Microscopic Findings

**Figure 4:**
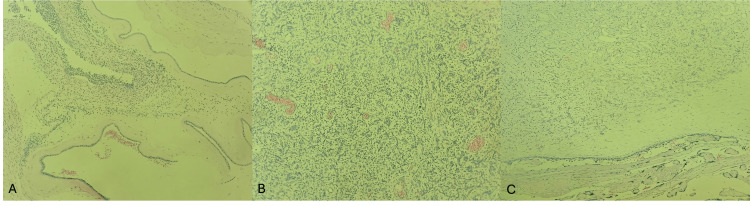
Histopathology images Microscopic pictures show (A) acute chorioamnionitis maternal inflammatory response stage 2; and (B-C) chorioangioma.

Microscopically, the placenta showed an 8 cm chorioangioma with areas of thrombosis, ischemic necrosis, and dystrophic calcifications, alongside organized subchorionic hematoma. There were features of acute necrotizing chorioamnionitis, predominantly localized to the site of membrane rupture. Along with the periphery of the tumor, clusters of avascular villi were identified, numbering no more than 20 per focus. Elsewhere in the placental parenchyma, the villi demonstrated stromal edema, increased cellularity, and focal hypervascularization. The umbilical cord contained a normal number and ratio of vessels. Histopathology images of the chorioamnionitis and the chorioangioma are demonstrated in Figure 4.

## Discussion

Large placental chorioangiomas, though typically benign, carry a high risk of maternal and fetal complications due to their vascular nature and potential to function as arteriovenous shunts. These lesions are detected antenatally on ultrasound with Doppler as well-circumscribed vascular masses protruding into the amniotic cavity, often adjacent to the umbilical cord insertion [11]. On MRI, they characteristically appear as well-defined, heterogeneous masses. On T1-weighted images, they are usually isointense to the placenta, although areas of high signal intensity may represent intratumoral hemorrhage. T2-weighted sequences frequently demonstrate a hyperintense and heterogeneous signal, reflecting the lesion’s vascular and cystic components. The detailed tissue characterization provided by MRI enhances diagnostic confidence and aids in distinguishing chorioangiomas from other placental masses [5,7,16].

Clinical manifestations of large chorioangiomas vary, but significant maternal and fetal morbidity is common. These tumors may lead to polyhydramnios, fetal anemia, hydrops fetalis, and preterm labor [17,18]. Maternal complications include postpartum hemorrhage, PPROM, preterm delivery, and preeclampsia [18,19]. Given the risk of fetal anemia, close surveillance is recommended every two to three weeks, with weekly assessments after 32 weeks of gestation. Monitoring typically includes regular ultrasound evaluation of tumor growth, fetal cardiac function, MCA PSV, and amniotic fluid volume as proposed in the literature, and many authors support this protocol [1,2].

Because of the rarity of large or symptomatic chorioangiomas, there is no universally accepted management protocol, and treatment must be individualized. Complex cases often require interventions aimed at mitigating the effects of arteriovenous shunting, whereas mild lesions may be managed with routine observation [14]. Therapeutic options include supportive measures such as intrauterine transfusion for anemia and amnioreduction for severe polyhydramnios [4,13]. More invasive, tumor-directed interventions typically reserved for significant or refractory cases include chemosclerosis, ultrasound-guided embolization, interstitial or fetoscopic-assisted laser ablation, and pharmacologic therapy with propranolol [3,9,10,20]. Prenatal propranolol for placental chorioangioma remains investigational. Evidence is limited to isolated case reports, dosing and selection criteria are not standardized, and potential fetal and neonatal risks include bradycardia, hypoglycemia, and growth restriction. Since fetal hemodynamics were preserved without anemia or hydrops, beta blockade was not indicated. The fact that the prognosis for big, symptomatic chorioangiomas is still uncertain despite these aggressive therapies highlights how serious the condition is [9,15].

Conservative management can be successful in carefully monitored cases without signs of significant shunting or anemia, as shown in recent case reports of giant chorioangiomas complicated only by polyhydramnios and managed expectantly with favorable outcomes [3]. The decision matrix depends on lesion size, evidence of fetal compromise (e.g., rising MCA PSV, hydrops, cardiomegaly), and the trajectory of amniotic fluid dynamics; close surveillance, including serial ultrasound with Doppler assessment of MCA PSV and cardiac function is critical to time interventions before irreversible injury occurs [12,14,18].

Furthermore, our case further highlights the risk of intrauterine infection, particularly chorioamnionitis, which can substantially contribute to maternal and neonatal morbidity and mortality and is frequently associated with preterm labor and neonatal sepsis. The interplay between polyhydramnios and intrauterine infection adds complexity to the clinical course and prognosis. Significant polyhydramnios and the attendant membrane overdistension create a proinflammatory and biomechanically compromised environment that predisposes to membrane weakening and rupture, setting the stage for intrauterine infection such as chorioamnionitis [21]. Mechanical stretch of the fetal membranes upregulates cytokines and matrix metalloproteinases, degrading the extracellular matrix integrity and initiating sterile inflammatory pathways that mirror infectious cascades, thereby lowering the threshold for microbial invasion and PPROM [22,23]. Chorioamnionitis, whether microbial or driven by sterile inflammation, is a well-established trigger of preterm labor and adverse neonatal outcomes, and its inflammatory cascade may be amplified by membrane stretch and altered amniotic dynamics in the setting of marked polyhydramnios [21-23]. This inflammation-driven crosstalk can be occult, with subclinical intra-amniotic inflammation accelerating deterioration before overt signs emerge. In this context, polyhydramnios in our patient may have reflected not only placental transudation but also created a milieu that increased susceptibility to chorioamnionitis through mechanical and immunologic perturbation of the membranes. Emphasizing this interaction adds important nuance to literature and underscores the need for vigilant surveillance in similar high-risk scenarios.

Notably, in our patient, the presence of significant polyhydramnios in the absence of elevated MCA PSV suggests that the excess amniotic fluid was not secondary to fetal anemia or high-output cardiac failure (conditions typically linked to increased fetal urine output). Instead, this finding supports the placental transudate hypothesis, which proposes that fluid accumulation results from passive transudation of serous fluid from the highly vascularized surface of the chorioangioma into the amniotic cavity [18,20]. Serial assessment of MCA PSV remained below 1.5 MoM throughout the pregnancy, suggesting preserved fetal hemodynamics.

The diagnosis of placental chorioangioma was definitively confirmed on histopathology, which revealed vascular proliferation within the placental tissue. Microscopically, these tumors are characterized by an angiomatous and cellular stroma, often with degenerative changes such as calcification; larger lesions may also show hyaline and myxoid degeneration [1,13,16]. Histopathological examination also demonstrated features of chorioamnionitis, indicating an underlying inflammatory process that likely influenced the clinical course.

This case underscores the importance of early detection, comprehensive fetal surveillance, and individualized management strategies in pregnancies complicated by large placental chorioangiomas. While intervention may be necessary in severe scenarios, careful conservative management can still yield favorable maternal and neonatal outcomes, even when the lesion is large and associated with high-risk features.

## Conclusions

In conclusion, chorioangiomas are benign placental tumors, but when they increase in size, they can lead to significant maternal and fetal complications. In our case, early detection and a comprehensive diagnostic workup with ultrasound and Doppler studies allowed the formulation of a conservative and vigilant management plan. Delivery timing must balance fetal maturity against the evolving risk of complications and should take place in a tertiary setting with immediate access to neonatal intensive care. Definitive diagnosis and exclusion of other placental lesions require postnatal histopathological examination. Because large symptomatic placental chorioangiomas are rare and often present with variable features, frequently in association with polyhydramnios, detailed case reports remain vital for advancing clinical understanding. This report aims to clarify the complex relationship between placental chorioangioma and polyhydramnios by outlining the diagnostic process, management decisions, and maternal and fetal outcomes. Moreover, it highlights the interplay between polyhydramnios and chorioamnionitis. Excess amniotic fluid and the resulting membrane overdistension can promote sterile inflammation and weaken the membranes, increasing susceptibility to infection, while intrauterine infection further destabilizes the pregnancy and may precipitate preterm labor. Sharing this experience emphasizes the importance of early diagnosis, close surveillance, and a multidisciplinary approach with the goal of informing future practice and improving perinatal outcomes.
